# Integrative Morphological, Physiological, Proteomics Analyses of Jujube Fruit Development Provide Insights Into Fruit Quality Domestication From Wild Jujube to Cultivated Jujube

**DOI:** 10.3389/fpls.2021.773825

**Published:** 2021-11-24

**Authors:** Jian Huang, Xin Chen, Aobing He, Zhibo Ma, Tianqi Gong, Kenong Xu, Ruihong Chen

**Affiliations:** ^1^Key Laboratory of National Forestry and Grassland Administration on Silviculture in Loess Plateau, College of Forestry, Northwest A&F University, Yangling, China; ^2^Horticulture Section, School of Integrative Plant Science, Cornell University, New York State Agricultural Experiment Station, Geneva, NY, United States; ^3^Key Laboratory of Shaanxi Province on Jujube, College of Life Science, Yan’an University, Yan’an, China

**Keywords:** *Ziziphus jujuba*, proteomics, fruit expansion, gibberellin, ascorbic acid

## Abstract

Jujube (*Ziziphus jujuba*) was domesticated from wild jujube (*Z. jujuba* var. *spinosa*). Here, integrative physiological, metabolomic, and comparative proteomic analyses were performed to investigate the fruit expansion and fruit taste components in a jujube cultivar ‘Junzao’ and a wild jujube ‘Qingjiansuanzao’ with contrasting fruit size and taste. We revealed that the duration of cell division and expansion largely determined the final fruit size, while the intercellular space in the mesocarp dictated the ratio of mesocarp volume in mature fruits. The high levels of endogenous gibbereline_3_ (GA) and zeatin in the growing fruit of ‘Junzao’ were associated with their increased fruit expansion. Compared with ‘Junzao,’ wild jujube accumulated lower sugars and higher organic acids. Furthermore, several protein co-expression modules and important member proteins correlated with fruit expansion, sugar synthesis, and ascorbic acid metabolism were identified. Among them, GA20OX involved in GA biosynthesis was identified as a key protein regulating fruit expansion, whereas sucrose-6-phosphate synthase (SPS) and neutral invertase (NINV) were considered as key enzymes promoting sugar accumulation and as major factors regulating the ratio of sucrose to hexose in jujube fruits, respectively. Moreover, the increase of Nicotinamide adenine dinucleotide-Malate dehydrogenase (NAD-MDH) activity and protein abundance were associated with the malic acid accumulation, and the high accumulation of ascorbic acid in wild jujube was correlated with the elevated abundance of GalDH, ZjAPXs, and MDHAR1, which are involved in the ascorbic acid biosynthesis and recycling pathways. Overall, these results deepened the understanding of mechanisms regulating fruit expansion and sugar/acids metabolisms in jujube fruit.

## Introduction

Chinese jujube (*Ziziphus jujuba* Mill.) is one of the major deciduous fruit crops and contributes greatly to economic development in rural regions in China (Liu et al., 2020). Jujube fruits have distinct nutritional values and a special flavor due to their high sugars (ca.72% of fruit dry biomass) and Vitamin C (over 1,000 mg per100 g fresh flesh) content. It is widely consumed over a long history in East Asia and has become increasingly popular in Western countries. Jujube is known to be domesticated from wild jujube (*Z. jujuba* Mill. var. *spinosa*), which resiliently evolved from fruits with a sour taste, small size, and thin pericarp to fruits with a sweet taste, large size, and high ratio of edible flesh (Guo et al., 2021). Since fruit development and ripening are processes regulated by both genetic and epigenetic factors, domestication must have remolded the genetic basis underlying the biological processes (Huang et al., 2016). Understanding the difference between wild jujube and cultivated jujube in fruit expansion and ripening would provide valuable information for the genetic improvement of jujube.

Domesticated fruit species, such as apple and pear, are often characterized with much enlarged fruit over their progenitor wild species. Similarly, compared with wild jujubes, cultivated jujubes also have significantly larger fruit (>10 g vs < 5 g) due to the high proportion of flesh tissues surrounding the stone. Fruit size, determined by the final cell number and cell volume, is strongly influenced by the strength and duration of the cell division and cell expansion phases (Malladi and Johnson, 2011). During the fruit set, growth, and ripening progress, plant hormones play a vital role in regulating each phase (McAtee et al., 2013; Kumar et al., 2014). Phytohormones including auxin, gibberellins (GAs), and cytokinins, can initiate fruit development, stimulate growth, and determine fruit size. Auxin promotes fruit set and growth, at least partly, by controlling the GA levels. For example, Auxin Response Factor (SlARF7) has been shown to mediate the crosstalk between auxin and GA (de Jong et al., 2011). Further, GA-mediated responses are regulated by a “relief of restraint” model, i.e., any activation of GA signaling requires the degradation of DELLA proteins (Sasaki et al., 2003). The application of these hormones alone or in combination will induce robust fruit set and rapid growth even in the absence of fertilization in jujube, prompting extensive hormone usage in jujube production in China. However, few studies, if any, were dedicated to the dynamics, synthesis, and signal transduction of endogenous phytohormones during fruit development in cultivated and wild jujubes.

The fruit taste is closely related to the composition and concentration of sugars and acids in the fruit. It is noted that the sugar content in jujube fruits is much higher than that in wild jujube and many other fruit tree species, such as apple, peach, and grape. Soluble sugars in fruits, including sucrose, fructose, and glucose, are not only essential for fruit growth as carbon sources but also central to fruit quality. Latest studies reported that the cultivated and wild jujubes exhibited a highly contrasting profile in their sugar and organic acid dynamics during fruit transition to ripening, including gradually accumulated sucrose and declined organic acids in cultivated jujubes and slightly elevated sucrose and unchanged high-level organic acids in wild jujubes (Huang et al., 2016; Song et al., 2019). Sugar contents in fruits are largely dependent on the balance between the sources and sink strength. Sucrose–The sucrose cycle is the center of sugar metabolism in the sink, which comprises the sucrose breakdown by invertase and sucrose synthase (SUSY), phosphorylation of the resultant hexoses, re-synthesis of sucrose *via* sucrose-6-phosphate synthase (SPS), and sucrose-6-phosphate phosphatase. Sucrose-Sucrose cycle links carbohydrates allocated from source to glycolysis followed by tricarboxylic acid (TCA) cycle, starch synthesis, and cellulose synthesis. It was revealed that the gene families involved in sugar metabolism were expanded more in the jujube genome than in the genomes of Rosales fruit (Liu et al., 2014). During jujube domestication, a series of genes involved in the key steps of sugar metabolism and organic acid metabolism had undergone a selection and their transcript levels were also more enhanced in cultivated jujubes than in wild jujubes (Huang et al., 2016). In addition, plasmodesmata are present in jujube cultivars, but absent in wild jujube, suggesting that sugar unloading systems differed between cultivated and wild jujubes as well. Moreover, sugar transporter genes were expressed highly during jujube fruit ripening, further elucidating the mechanism of high sugar accumulation in jujube fruits (Zhang et al., 2016). However, there is still a lack of knowledge on how the balance between sugar and organic acid, and sugar composition are regulated in cultivated and wild jujubes.

Although previous studies have characterized jujube fruit development and taste at the genomics and transcriptional levels, it is not sufficient to describe the molecular mechanisms underlying fruit expansion and taste development due to the wider dynamic range in protein than in transcript abundance and poor correlations between messenger RNA (mRNA) and protein levels. Recent advances in liquid chromatography with tandem mass spectrometry (HPLC-MS/MS)-based high-throughput proteomics technologies have made it feasible to identify a large number of proteins in the tissues, organs, and cells to elucidate the molecular mechanisms of fruit development and ripening in fruit species (Palma et al., 2011), such as apple (Li et al., 2016), citrus (Katz et al., 2011), pear (Wu et al., 2014), and kiwifruit (Tian et al., 2021). In these studies, several key steps in controlling sugars/acids accumulation, fruit ripening, and flavor development were identified, deepening the understanding of the biological progress of fruit development. Based on the assembled jujube genome (Huang et al., 2016), it is possible to perform a reliable proteomic analysis of jujube fruit against the self-built protein database deduced from the genome annotations. In this study, we carried out a comparative proteomic analysis that was integrated with phytohormones and enzyme activity analyses, as well as histological observations between a cultivated jujube cultivar and a wild jujube accession at different stages of fruit development to identify the key regulators of fruit expansion and the metabolism of sugars and acids.

## Materials and Methods

### Plant Material

We selected a cultivated jujube cultivar ‘Junzao’ and a wild jujube accession ‘Qingjiansuanzao’ in the Jujube Experimental Station of Northwest A&F University (Qingjian, Shaanxi, China; N 37.13, E 110.09) in 2016, which have been used in the previous genome sequencing project (Huang et al., 2016). Fruit samples were harvested at five development stages, i.e., young [20 days after flowering (DAF)], enlarging (40 DAF), white mature (60 DAF), beginning red (80 DAF), and fully red (100 DAF), from 10-year-old trees of both ‘Junzao’ and ‘Qingjiansuanzao’ At least 10 fruits were randomly collected from one plant as a biological replicate, and three replicates were prepared. The harvested fruits for the proteomics and physiological analysis were immediately cut into small pieces and rinsed in liquid nitrogen, and then transported to the laboratory. All samples were stored at −80°C before use.

### Anatomy Observation of Flesh Tissue

The fruits of the two accessions were sampled every week from 7-DAF to 49-DAF, and an additional sampling at 77-DAF was also conducted. The sampled fruits were sliced transversely in the field and immediately rinsed in a formalin acetic acid (FAA) solution [50% ethanol, 5:5:90, v/v/v], and then vacuum infiltrated and fixed for 24 h. The samples were dehydrated and stained with Schiff’s reagent-fast green. The sections were observed under a digital optical microscope (Olympus BX43, Tokyo, Japan). The sizes of parenchymal cells in the pericarp were determined by measuring the lengths of the long and short axes using the associated software “Cellsense Standard.” The average cell area was determined in three fields in each of two sectors per fruit, and the intercellular space was also calculated by the rest space. The number of cell layers was counted from the endocarp to the exocarp in at least three independent sectors.

### Determination of Soluble Sugars, Organic Acids Content During Fruit Development

Soluble sugar determination was performed as described previously (Gao et al., 2012). Briefly, flesh samples were homogenized in 1 ml mobile phase [acetonitrile: H_2_O (8:2, v/v)] and extracted by an ultrasonication method. the Glucose, fructose, and sucrose were analyzed on a Hitachi L2000 high-performance liquid chromatography (HPLC) system equipped with a COSMOSIL Packed Column 5NH2-MS (4.6 mm I.D × 150 mm) and detected by a refractive index detector (L-2490, Merck-Hitachi, Tokyo, Japan). The solution was extracted for organic acids, including malic acid, citric acid, succinic acid, and quinic acid, with a similar method except that the extraction solution was replaced with 1 ml0.05 M KH_2_PO_4_ (pH 2.4), and determined on the HPLC system equipped with ECOSIL HPLC C18 Column (4.6 × 250 mm) and L-2400 UV Detector.

### Determination of Endogenous Phytohormones in Fruits

Endogenous phytohormones were determined by an HPLC-MS method as described in Pan et al. (2010). Approximately 0.5 g of flesh sample was ground in liquid nitrogen, and homogenized with a solvent [methanol: deionized water: formic acid (15:4:1, v/v/v)] assisted by ultrasonication (power: 250 W) for 30 min. The samples were separated by an HPLC system (LC−20AT, SHIMADZU Co. Ltd., Kyoto, Japan) equipped with an Agilent Eclipse XDB-C18 column (2.1 × 150 mm, 5 μm, United States). Mass spectrometric detection was carried out on AB SCIEX QTRAP 5500 MS (United States) under optimized conditions.

### Enzyme Activity Assays

The enzyme was extracted from the fruit flesh tissue and activities of the acid invertase (AI), neutral invertase (NI), sucrose synthase (SS), and sucrose phosphate synthase (SPS) were determined as described in Hubbard et al. (1989). Amylase activity determination was performed according to (Tian et al., 2021). Citrate synthase, Cytoplasm-Aconitase (Cyt-ACO), Nicotinamide adenine dinucleotide-Malate dehydrogenase (NAD-MDH), and NADP-Malic enzyme (NADP-ME) were extracted and determined as described in Hirai and Ueno (1977).

### Proteomics Analysis

At first, 2 g of mixed fruit flesh samples from one plant were ground into fine powder in liquid nitrogen and homogenized in 10 ml trichloroacetic acid: acetone (1:9, V/V) solution, and proteins were extracted according to a method described previously (Chen et al., 2017). The extracted protein concentration was determined using a bicinchoninic acid protein assay kit (BCA, Beyotime, China) and adjusted to 5 mg/ml. To determine integrity and quality in each sample, 20 μg of the protein extract was separated by electrophoresis on a 12.5% sodium dodecyl sulfate-polyacrylamide gel and was stained with Coomassie Brilliant Blue staining buffer (GelCode blue; Pierce, Waltham, MA, United States). The digestion of proteins (300 μg protein extracts) was conducted using the filter-aided sample preparation (FASP) procedure as described in Wisniewski and Rakus (2014). The obtained peptide content was estimated using a UV light spectral density at 280 nm using an extinction coefficient of 1.1 of0.1% (g/l) solution that was calculated based on the frequency of the tryptophan and tyrosine in vertebrate proteins.

Mass spectrometry analyses were performed on a Q Exactive mass spectrometer that was coupled with an Easy nLC 1000 (Proxeon Biosystems, Thermo Fisher Scientific, Waltham, MA, United States). Five micrograms (5 μg) peptide was loaded onto a C18 reversed-phase column (Thermo Scientific Easy Column, 10 cm long, 75 μm inner diameter, 3μm resin) in buffer A (2% acetonitrile and0.1% Formic acid) and separated with a linear gradient of buffer B (84% acetonitrile and0.1% Formic acid) at a flow rate of 400 nl/min controlled by IntelliFlow technology for 120 min. The linear gradient was determined by 2 h gradient:0–45% buffer B for100 min, 45–100% buffer B for 8 min, and hold in 100% buffer B for 12 min. Mass spectrometry data were acquired using a data-dependent top 20 method that dynamically chooses the most abundant precursor ions from the survey scan (300–1,800 m/z) for high-energy collision dissociation (HCD) fragmentation. The determination of the target value was based on a predictive Automatic Gain Control. The dynamic exclusion duration was 25 s. Survey scans were acquired at a resolution of 70,000 at m/z 200, and resolution for HCD spectra was set to 17,500 at m/z 200. The normalized collision energy was 30 eV, and the underfill ratio, which specifies the minimum percentage of the target value likely to be reached at maximum fill time, was defined as 0.1%. The instrument was run with peptide recognition mode enabled. The mass spectrometry (MS) experiments were performed three times for each sample.

### Sequence Database Searching and Peptide Identification

The MS raw data were analyzed using MaxQuant software (1.5.3.17) (Cox and Mann, 2008), and processed. The MS data were searched against a self-built database (27,443 total entries) generated from the *Z. jujuba* ‘Junzao’ genome sequence (National Center for Biotechnology Information (NCBI) accession LPXJ00000000). The parameters in the MaxQuant searches were set as follows: Carbamidomethylation of cysteine was defined as a fixed modification, and oxidation of methionine and protein N-terminal acetylation were set as a variable modification. An initial search was set at a precursor mass window of 6 ppm, and two missed cleavage sites were allowed. Both the first search and MS/MS mass tolerance were set as 20 ppm for fragment ions. The false discovery rate (FDR) at the peptide and protein level was determined by searching the same data set against the target database and a decoy database. Only peptides with confidence >99% and false discovery rate <0.01 were considered as identified proteins. Each protein was provided with a confidence score based on the confidence scores of its peptides with unique spectral patterns. Protein abundance was calculated based on the normalized spectral protein intensity (LFQ intensity) (Cox et al., 2014). To compare the protein abundances across different samples, we used the label-free quantification (LFQ) algorithm, which compares the intensities of the same peptides detected in different samples (Cox et al., 2014). Only the data with at least two values of the three replicates be used to perform a *t*-test. A twofold cut-off alongside *p* < 0.05 was used to declare the quantitative changes of the up- and down-regulated proteins.

### Statistical Analysis and Bioinformatics Analysis

The physiological data were analyzed using one-way ANOVA, followed by Duncan’s multiple range tests. The Pearson correlation coefficient (*r*) and probability (*p*) between sugars, organic acids concentrations, and enzyme activities were also analyzed. The correlation and ANOVA analyses were performed using SPSS 18.0 (IBM, Armonk, NY, United States).

The protein abundance data were subjected to cluster analysis based on a hierarchical clustering method with Euclidean distance and average linkage using Cluster 3.0 (de Hoon et al., 2004) and were displayed in Java Treeview (Saldanha, 2004) to examine the repeatability and quality of Label-free analyses globally. We used a twofold cut-off between different groups and *p* < 0.05 to designate changes in abundance as significant for the regulated proteins. To characterize the protein co-expression network related to fruit development, a weighted gene co-expression network analysis (WGCNA) was performed on the proteome databases using the WGCNA package version 1.6.6 in R (Langfelder and Horvath, 2008). The modules were obtained using the automatic network construction function blockwise Modules with default settings, with minor modifications (the power was 6, TOM-Type was adjacency, minModuleSize was 30, and mergeCutHeight was0.20). The eigengene value was calculated for each module and used to test the association with each stage of two accessions. The total connectivity, intramodular connectivity, and kME *P*-value were calculated. The hub genes in each module were defined by kME > 0.95, which measures the connectivity of a gene in the specific module. To characterize those modules, Kyoto Encyclopedia of Genes and Genomes (KEGG) pathways were performed in TBtools version1.068 (Chen et al., 2020) with a *P*-value <0.05. The resulting modules were represented and visualized as graph networks by Cytoscape 3.5.1^1^ using a circular layout to visualize the various inter-connections identified in the datasets. The corresponding RNA-sequencing data of two accessions were retrieved from our genome-sequencing project and were used for analyzing the relationships between the protein abundance and the transcriptional level.

## Results

### Fruit Growth Patterns in Cultivated and Wild Jujubes

**FIGURE 1:**
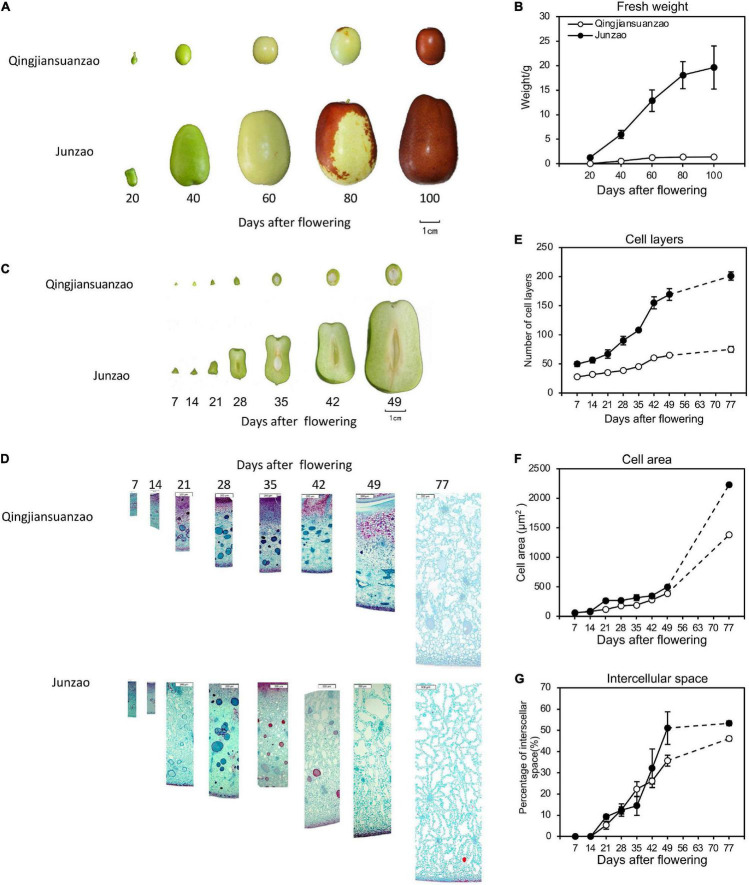
The fruit growth pattern of cultivated jujube ‘Junzao’ and wild jujube ‘Qingjiansuanzao’. **(A)** Fruit ripening progress of ‘Junzao’ and ‘Qingjiansuanzao’ represented by five stages at 20, 40, 60,80, and 100 days after flowering (DAF); **(B)** Fruit fresh weight increase patterns; **(C)** Cross-section of fruits sampled weekly from 7 DAF to 49 DAF; **(D)** Anatomy observation of the mesocarp from 7 to 49 DAF and 77 DAF; **(E–G)** indicate the change in the pattern of the cell layer, cell area, and intercellular space of the mesocarp from 7 to 49 DAF weekly and at 77 DAF. Values are means of three biological replicates ± SD.

Reflected by fruit size and weight, the cultivated jujube ‘Junzao’ fruit expanded rapidly before 80 DAF. In contrast, the wild jujube ‘Qingjiansuanzao’ fruit increased rapidly before 40 DAF and almost ceased at 60 DAF. The ‘Junzao’ fruit displayed an obviously larger size and heavier weight than the ‘Qingjiansuanzao’ fruit from 20 DAF and onward. At 100 DAF, the ‘Junzao’ fruit was nearly 5 times of ‘Qingjiansuanzao’ in size and about 14 times in weight (19.6 g vs 1.4 g) (Figures 1A–C). Longitudinal fruit sections showed that the fruit mesocarp underwent rapid growth from 14 DAF in ‘Junzao’ while it expanded slowly in ‘Qingjiansuanzao’; however, the fruit stone of both accessions displayed a similar transverse diameter before 49 DAF. The ratio of ‘Junzao’/‘Qingjiansuanzao’ in pulp radius increased from 1.6 at 7 DAF to 5 at 49 DAF. Thus, the mesocarp expansion led to a striking difference in the final fruit size between the two jujubes. The ratio of pulp/stone in transverse diameter decreased gradually in the wild jujube while it increased in ‘Junzao’ (Figure 1C). Based on anatomy observation, cells of mesocarp divided rapidly from 7 DAF to 49 DAF and maintained in ‘Junzao’ in subsequent stages, while cell layers increased slowly and nearly stopped after 49 DAF in ‘Qingjiansuanzao’. Finally, the mesocarp cell layers of ‘Junzao’ were 2.7 times those of ‘Qingjiansuanzao’ at 77 DAF (Figures 1D,E). On the other hand, the cell expanded slowly in both accessions before 49 DAF and expanded quickly to 77 DAF, and the cell area of the ‘Junzao’ mesocarp was 1.6 times more than that of ‘Qingjiansuanzao’ at 77 DAF (Figure 1F). Stony endocarp was developed at 21 DAF in ‘Qingjiansuanzao’, while it was developed beginning at 28 DAF in ‘Junzao’ (Figure 1D). Notably, intercellular space was visible at 21 DAF among the flesh cells and expanded by over 45% of the mesocarp volume at 77 DAF, leading to fruit cracks when it rains (Figure 1G). Thus, the difference in the duration and strength of cell division and expansion contributed to the final difference in fruit size between the cultivated and wild jujubes.

### Phytohormones Dynamics During Jujube Fruit Development and Ripening

**FIGURE 2:**
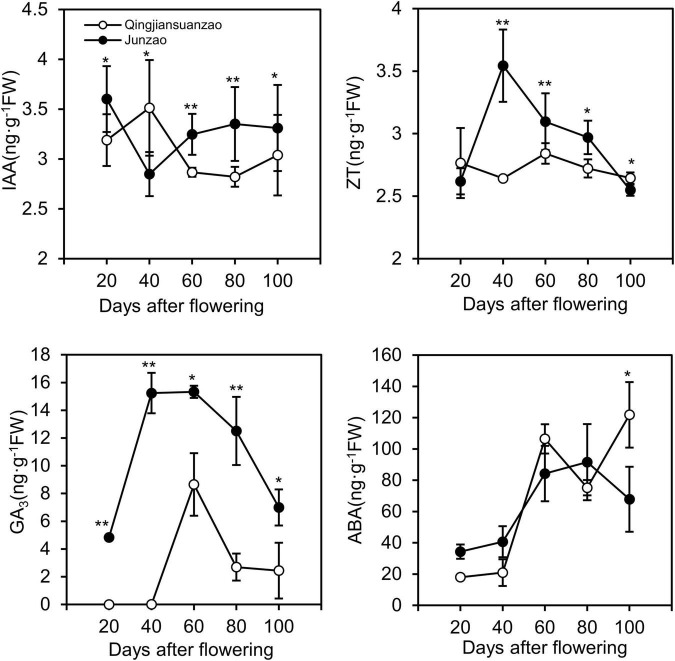
Dynamics of phytohormones IAA, ZT, GA_3_, and ABA during fruit development and ripening in jujube cultivar ‘Junzao’ and wild jujube ‘Qingjiansuanzao’. Values are means of three biological replicates ± SD. * and ** indicate significant differences at levels of *p* < 0.05 and *p* < 0.01 by *t*-test, respectively.

The contents of Gibberellin A3 (GA_3_), Zeatin (ZT), Auxin (IAA), and abscisic acid (ABA) were detected in fruits of both accessions at the five stages. As shown in Figure 2, the contents of GA_3_ and ZT peaked at 40–60 DAF in ‘Junzao,’ and 60 DAF in ‘Qingjiansuanzao’. The fruit accumulated more GA_3_ in ‘Junzao’ than in ‘Qingjiansuanzao’ at all stages. The concentration of ZT peaked at 40 DAF in ‘Junzao’ and 20 DAF in ‘Qingjiansuanzao’. The content of ZT was significantly higher in ‘Junzao’ than in ‘Qingjiansuanzao’ during 40–100 DAF. The content of IAA fluctuated and changed slightly during the fruit development of both accessions, and it presented a higher level in ‘Junzao’ during 60–100 DAF. The ABA content was maintained at low levels at 20- and 40-DAF, and was markedly increased to a high level at 60 DAF. In general, the hormones IAA, GA_3_, and ZT in the flesh were kept at high levels during 20–60 DAF and decreased in later stages. However, the content of ABA was maintained at a low level during 20–40 DAF and rapidly increased at 60 DAF, suggesting that fruit ripening likely initiates around 60 DAF.

### Changes in Sugar and Organic Acids and Related Enzymes Activities During Fruit Ripening

**FIGURE 3:**
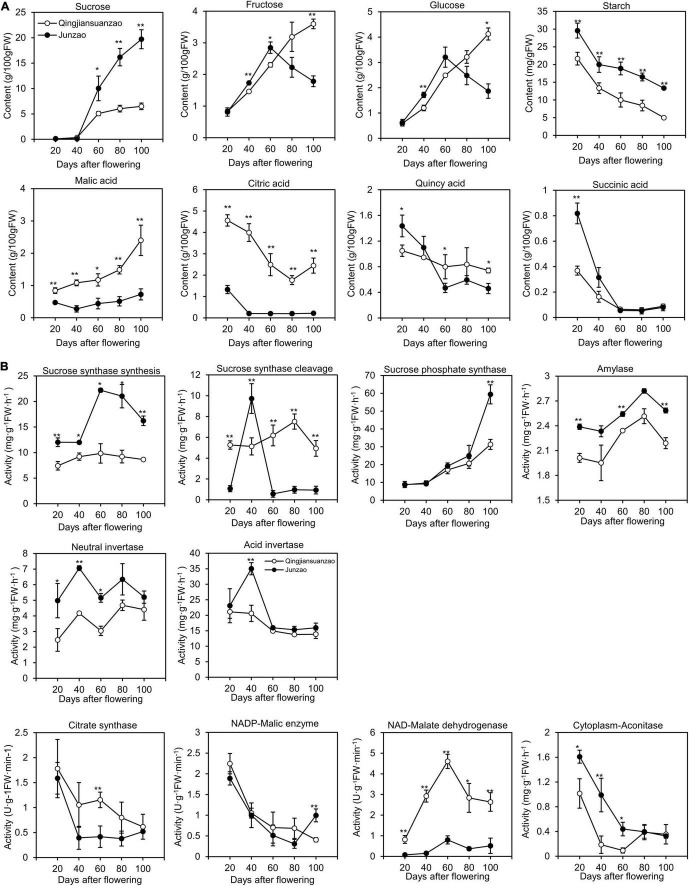
Dynamics of sugar, organic acids, and related enzyme activities during fruit development and ripening. **(A)** Changes in contents of sucrose, fructose, glucose, malic acid, citric acid, quincy acid, and succinic acid during fruit development in ‘Junzao’ and ‘Qingjiansuanzao’; **(B)** Activity changes of key enzymes involved in sugar and organic acid metabolism. Values are means of three biological replicates ± SD. * and ** indicate significant differences between the two accessions at levels *p* < 0.05 and *p* < 0.01 by *t*-test, respectively.

There were no significant differences in sucrose content between the two accessions during the early stages (20- and 40-DAF). However, the content of sucrose increased dramatically after 40 DAF in ‘Junzao’ and reached the maximum [20 g/100 g fresh weight (FW)] at 100 DAF, while it was almost plateaued (5 g/100 g FW) at 60 DAF in ‘Qingjiansuanzao’ (Figure 3). The levels of fructose and glucose peaked at 60 DAF and then decreased in ‘Junzao’ in later stages, while they gradually increased in ‘Qingjiansuanzao’ through 100 DAF. Therefore, sugar/glucose or fructose switched at 60 DAF in both accessions. Finally, the contents of both fructose and glucose in ‘Qingjiansuanzao’ were about two times higher than those in ‘Junzao.’ The ratio of sucrose to glucose/fructose was 10.5/10.9 in ‘Junzao’ and much greater than 1.6/1.8 in ‘Qingjiansuanzao’. The starch contents decreased gradually alongside fruit development and ripening in both accessions. For example, the starch content of ‘Junzao’ was 29.5 mg/g at 20 DAF and 13.4 mg/g in the ripened fruit, while the corresponding starch contents were 21.7 and 5.1 mg/g, respectively, in ‘Qingjiansuanzao’. Thus, sucrose was the major sugar in both accessions, although the sucrose vs. hexose ratio was much higher in ‘Junzao’ than in ‘Qingjiansuanzao’.

The activities of enzymes acid invertase (AINV), neutral invertase (NINV), Amylase, SS, and were higher in ‘Junzao’ than in ‘Qingjiansuanzao’ at all stages (Figure 3). The SS activity in the synthesis direction was elevated to a higher level from 40 to 60 DAF in ‘Junzao,’ but it was only slightly increased from 20 to 60 DAF in ‘Qingjiansuanzao’. The SPS activity increased gradually along with fruit ripening in both accessions.

In the cleavage direction, the SS activity peaked abruptly at 40 DAF in ‘Junzao.’ Interestingly, AINV and NINV also showed their activity peaks at this stage. The SS cleavage (SSc) activity in ‘Qingjiansuanzao’ was generally higher than in ‘Junzao’ although the AINV activity decreased slowly at 40 DAF and the NINV activity elevated to a higher level at two late stages. The SS (synthesis, *r* = 0.664, *p* < 0.01) and SPS (*r* = 0.843, *p* < 0.01) were strongly correlated with sucrose contents. The SSc and NINV activities were positively correlated with fructose and glucose contents. These data indicated that SSs and SPS contributed to the sugar accumulation while SSc and NINV might have regulated the sugar composition.

The quantification of four organic acids (malic acid, citric acid, quinic acid, and succinic acid) during fruit development and ripening showed that the cultivated and wild jujubes had a similar trend of variation, i.e., the contents of citric acid, quinic acid, and succinic acid gradually decreased while the levels of malic acid increased (Figure 3A). However, the two accessions also differed considerably in specific organic acid contents. In wild jujube, citric acid and malic acid were the dominant organic acids, which had similar contents (ca. 2.4 g/100 g) in the full mature stage. In cultivated jujube ‘Junzao’, the contents of quinic acid and malic acid were about 0.7 g/100 g, higher than the contents of citric (0.22 g/100 g) and succinic acid (0.08 g/100 g).

The activities of enzymes Citrate synthase (CS), Cyt-ACO, and NADP-ME decreased generally during fruit development (Figure 3B). The activity of NAD-MDH, which was consistently higher in ‘Qingjiansuanzao’ than in ‘Junzao,’ peaked at 60 DAF and then decreased slightly. Moreover, citric acid contents were positively correlated with the activities of CS in both directions (*r* = 0.73, *p* < 0.01), and malic acid concentration was positively correlated with the activities of NAD-MDH (*r* = 0.64, *p* < 0.01). These results indicated that NAD-MDH is likely one of the key enzymes regulating malic acid accumulation, and CS might have played a major role in citric acid accumulation.

### Proteomic Profiling and Protein Co-expression Networks Correlated With Fruit Development

**FIGURE 4:**
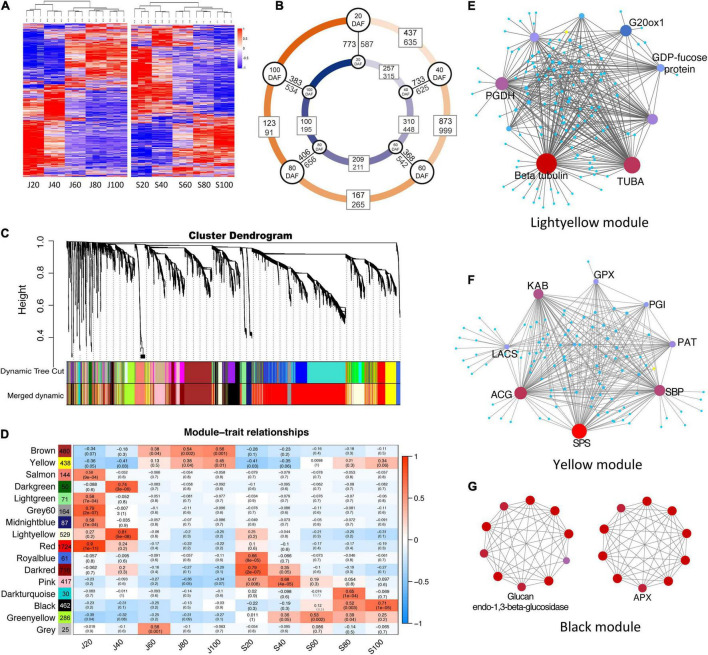
Proteomic profile and protein co-expression network analysis during the fruit development of ‘Junzao’ and ‘Qingjiansuanzao’. **(A)** Heatmap representation of protein expression profile during fruit development. J20–J100 and S20–S100 represent 20, 40, 60, 80, and 100 DAF of ‘Junzao’ and ‘Qingjiansuanzao,’ respectively. **(B)** Number of differential expressed proteins (DEPs) detected between neighbor stages and between ‘Junzao’ (indicated by a blue inner circle) and ‘Qingjiansuanzao’ (orange outer circle) at the same stage. **(C)** Hierarchical cluster tree of co-expression modules identified by weighted gene co-expression network analysis (WGCNA). Each leaf in the tree represents one protein and each tree branch stands for a module. **(D)** Module–fruit developmental stage associations. The color of each cell at the row–column intersection indicates the correlation coefficient *r* (up) between the modules (row) and the protein expression profile at a fruit developmental stage (column) and the r-associated significance *p*-value (down). The WGCNA network of top hub genes in modules Yellow **(E)**, Lightyellow **(F)**, and Black **(G)**. PGDH, D-3-phosphoglycerate dehydrogenase; ACG, glucose-6-phosphate 1-dehydrogenase; SBP, selenium-binding protein 2; SPS, sucrose-phosphate synthase; KAB, voltage-gated potassium channel; PAT, bifunctional aspartate aminotransferase and glutamate; GPX6, phospholipid hydroperoxide glutathione peroxidase 6; LACS2, long chain acyl-CoA synthetase 2; PGIC1, glucose-6-phosphate isomerase; ALDH3H1, aldehyde dehydrogenase family 3 member H1; CCR1, cinnamoyl-CoA reductase 1; APX, ascorbate peroxidase.

A total of 5,380 proteins were identified in the fruit samples studied (Supplementary Table 1), accounting for 26.14% of the protein-encoding genes detected in their corresponding transcriptomes (accession SRX1518646-56). Among them, 4,998 and 4,797 proteins were identified in ‘Junzao’ and ‘Qingjiansuanzao,’ respectively, and 4,616 proteins were overlapped between the two datasets (Figure 4A). Among these proteins, 1,072, 1,572, 432, and 214 were differentially expressed proteins (DEPs) detected during the periods of 20–40, 40–60, 60–80, and 80–100 DAFs, respectively. During the corresponding periods, there were fewer DEPs identified in ‘Qingjiansuanzao’ than in ‘Junzao,’ which were 572, 758, 420, and 295 proteins, respectively (Figure 4B). In addition, we identified 1,360, 1,358, 910, 1,062, and 917 DEPs between the two accessions at the five stages of 20, 40, 60, 80, and 100 DAFs, respectively (Figure 4B). Clearly, there were many more DEPs between the two accessions in 20- and 40-DAF than in 80- and 100-DAF. These results suggested that the fruit proteomes were regulated more intensively before 60 DAF in both accessions and that stage 60 DAF was likely the reprogrammed phase of the proteome during jujube fruit developing and ripening.

Protein co-expression networks analysis identified 15 distinct WGCNA modules correlated with the protein expression profile in at least one developmental stage in the two accessions (Figures 4C,D). The protein numbers in each module ranged from 30 in the Darkturquoise module to 1,724 in the red module. Among the different developmental stages, J20 (20 DAF of ‘Junzao’) showed a protein expression profile that was correlated with the most (seven) number of modules (Yellow, Salmon, Lightgreen, Grey60, Midlightblue, Red, Greenyellow). However, J60 and S60 (60 DAF of ‘Junzao’ and ‘Qingjiansuanzao’, respectively) were only correlated with one module, while other stages were correlated with two or three modules (Figure 4D). In addition, modules of Brown and Yellow showed an increased correlation significance from J80 to J100 during fruit ripening in ‘Junzao,’ while a similar correlation trend in ‘Qingjiansuanzao’ was observed only on the Black module.

The KEGG pathway analyses of the member proteins in the modules (Supplementary Figure 1) identified several enriched pathways. In the modules positively correlated with J20 and S20, the enriched pathways included genetic information processing, translation, and mRNA-related pathways. Notably, the number of proteins enriched in these KEGG pathways was far more in J20 than in S20. For example, the number of enriched proteins in the pathway of genetic information processing was 500 in J20 vs. 15 in S20. At 40 DAF, the enriched pathways included amino sugar and nucleotide sugar metabolism, starch and sucrose metabolism, and ascorbate and aldarate metabolism in ‘Junzao,’ while the pathways of sucrose metabolism and ascorbate metabolism were not enriched in ‘Qingjiansuanzao’. Interestingly, the pathways of carbohydrate metabolism and energy metabolism were enriched early from S20 to S60 in ‘Qingjiansuanzao’ while they were enriched late in stages J60–J100 in ‘Junzao.’ During 60–100 DAF, the pathways of carbohydrate metabolism, energy metabolism, amino acid metabolism, pyruvate metabolism, and particularly vitamin metabolism became enriched more significantly in ‘Qingjiansuanzao’ than in ‘Junzao.’ Such differential pathway enrichments appeared to suggest that the pathways of cell division, sucrose metabolism, and ascorbate metabolism in earlier stages were more active in ‘Junzao’ while the pathways of carbohydrate metabolism and energy metabolism were more active in ‘Qingjiansuanzao’, presumably contributing to the different features in fruit development and ripening between the two accessions.

We further examined the intra-modular connectivity of the hub proteins (Supplementary Table 2) and uncovered the following: (1) In modules Grey60 (*r* = 0.79, *p* = 2e-07) and Salmon (*r* = 0.58, *p* = 9e-04) that were positively correlated with J20, ribosomal proteins (Zj.jz013737024, Zj.jz014397089) in module Grey60, and cell growth-regulating nucleolar protein (Zj.jz017079171), cell division cycle and apoptosis regulator protein (Zj.jz022467015), tubulin (Zj.jz043343304), and DNA polymerase (Zj.jz044451020) in module Salmon showed higher connectivity in ‘Junzao’ than in ‘Qingjiansuanzao’. (2) In module Lightyellow, which was correlated with J40 (*r* = 0.81, *p* = 5e-08), tubulins (Zj.jz019903023, Zj.jz041961060) were the highest connected member proteins (Figure 4E), suggesting cell division remained more active in ‘Junzao’ than in ‘Qingjiansuanzao’ at DAF 40. For module Yellow, its correlation increased from 80 DAF (*r* = 0.38, *p* = 0.04) to 100 DAF (*r* = 0.45, *p* = 0.01) in Junzao.” In this module, the member protein sucrose-phosphate synthase (Zj.jz019851046) was identified as a hub protein with the second-highest connectivity (46 proteins) (Figure 4F). (3) In the Pink module that was correlated with S40 (*r* = 0.68, *p* = 4e^–5^), the member proteins phosphoglycerate kinase (Zj.jz024217060, PK) and glyceraldehyde-3-phosphate dehydrogenase (Zj.jz034129113) had higher connectivity (11 and 6 proteins), indicating that glycoses was more active at DAF 40 in ‘Qingjiansuanzao’ than in ‘Junzao.’ In module Black that was correlated with S80 (*r* = 0.52, *p* = 0.003) and S100 (*r* = 0.71, *p* = 1e-05), the fruit soften related protein Glucan endo-1,3beta-glucosidase (Zj.jz014397075) and the ascorbate acid oxidation related protein L-ascorbate peroxidase (Zj.jz020529001) showed higher connectivity (7 proteins) (Figure 4G). Overall, these findings suggest that the pathways in which the hub proteins are involved likely represent an important signature for fruit expansion, fruit ripening, and sugar accumulation in ‘Junzao’ and ‘Qingjiansuanzao’.

### Proteins Involved in Phytohormone Metabolisms, Cell Division, and Cell Expansion

**FIGURE 5:**
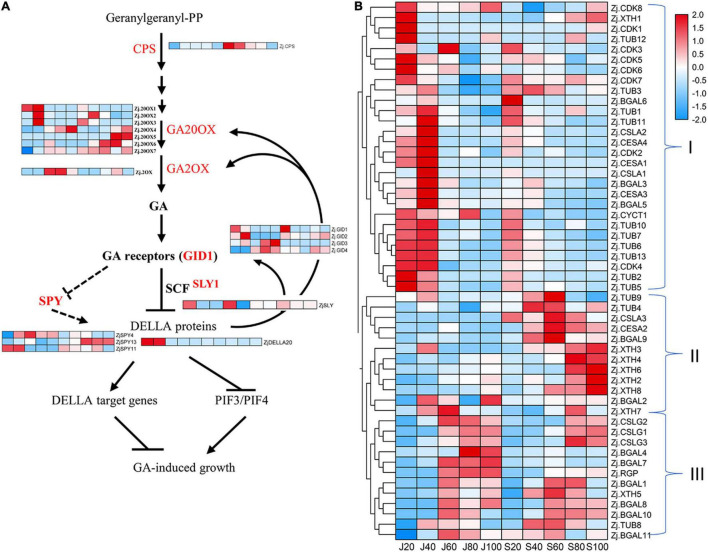
Accumulation profile of proteins associated with gibberellin (GA) and cell division and expansion. **(A)** Expression of identified proteins involved in GA biosynthesis and signal transduction. **(B)** Expression pattern of proteins related to cell production and cell wall modification.

As described in section 2.2 above, GA_3_, IAA, and ZT likely played important roles in stimulating jujube fruit expansion. Seventeen proteins were involved in GA synthesis and signal transduction, including seven ZjG20OXs, five GA receptors, four Gibberellins receptors (ZjGID), three SPY, and one each for proteins ZjCPS, ZjG2OX, SLY, and DELLA (Figure 5A and Supplementary Table 3). In general, proteins involved in GA synthesis were more abundant in ‘Junzao’ than in ‘Qingjiansuanzao’ across the five stages. The proteins ZjG20OX1,2,3 were highly expressed before 60 DAF and were downregulated to low levels (Figure 5A). The ZjG2OX (Zj.jz039269024) expression was upregulated during 40–80 DAF, consistent with the GA_3_ pattern of change during fruit development. We considered that these G20OXs were the key proteins regulating GA synthesis and high expression levels would lead to high contents of GA_3_ in ‘Junzao.’ Similarly, GA receptors ZjGID1,2 were highly expressed during 20–40 DAF and were more abundant in ‘Junzao.’ Both ZjSLY and ZjDELLA20 proteins were expressed highly in the early stages. We identified a cytokinin hydroxylase (ZjCYP735A, Zj.jz024411042), which showed a decreasing trend and became undetectable at 60 DAF in ‘Junzao’ and 40 DAF in ‘Qingjiansuanzao’. We also identified three proteins ZjTAA3 (Zj.jz002305049), ZjYUCCA1 (Zj.jz001627099), andZjGH3.7 (Zj.jz043973001) involved in IAA synthesis and signal transduction, which showed an increasing trend during fruit ripening. Among them, ZjTAA3 and ZjYUCCA1 were more abundant in ‘Junzao’ than in ‘Qingjiansuanzao’. These results suggested that the synthesis of GA, ZT, and GA signal transduction were more active in ‘Junzao’ than in ‘Qingjiansuanzao’.

Moreover, we identified 53 proteins involved in cell cycle, cell wall construction, and modification, including 11 β-galactosidases (ZjBGALs), three Cellulose synthase-like proteins (ZjCSLGs), eight Cyclin-dependent kinases (ZjCDKs), 13 Tubulins (ZjTUBs), eight xyloglucan endotransglucosylase/hydrolase (ZjXTHs), three mannan synthases/transferases (ZjCSLAs), four cellulose synthases/transferases (ZjCESAs), and three others (Supplementary Table 3). Based on their expression abundance and change patterns, the 53 proteins were clustered into three groups (Figure 5B). Group I was clustered mostly with proteins related to cell production and expansion, such as ZjTUBs, ZjCYC, ZjCDKs, ZjCSLAs, and ZjCESAs. Most of them were expressed highly during 20–40 DAF and were downregulated at 60 DAF. Their abundance was generally higher in ‘Junzao’ in young stages. We considered that these proteins may take part in fruit expansion closely. Most proteins in Group II were upregulated during 40–80 DAF or 80–100 DAF in ‘Qingjiansuanzao’. Among them, we found five ZjXTHs that were highly expressed during 80–100 DAF in ‘Qingjiansuanzao’ while constantly at low levels in ‘Junzao.’ Since fruit softening occurred earlier in wild jujube, the ZjXTHs might have played a role in fruit softening. In Group III, most proteins showed a similar trend and were highly expressed during 60–100 DAF in both accessions.

### Differentially Expressed Proteins Involved in Sugar and Organic Acid Metabolism

**FIGURE 6:**
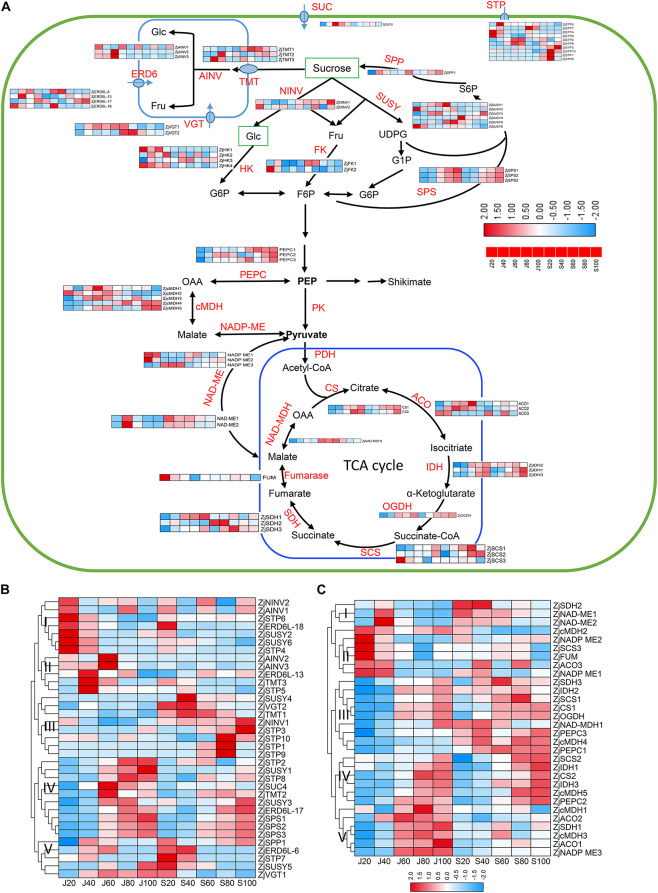
Expression profile of proteins involved in sugar and organic acid metabolism. **(A)** Pathway of sugar metabolism and transport in jujube fruit. **(B)** Expression profile of proteins related to sugar metabolism and transport in the fruits of ‘Junzao’ and ‘Qingjiansuanzao’ from 20 DAF to 100 DAF. **(C)** Expression profile of proteins involved in organic acid metabolism related to glycolysis and TCA cycle in the fruits of ‘Junzao’ and ‘Qingjiansuanzao’ from 20 DAF to 100 DAF . Note that only a subset of primary proteins required for soluble sugar and starch metabolism and transportation in jujube fruits is displayed. The heatmaps were drawn using log_2_-transformed protein abundance. Abbreviations of enzymes for sugar metabolism and transportation: FK, fructokinase; F6P, fructose-6-phosphate; Fru, D-fructose; Glc, D-glucose; G6P, glucose-6-phosphate; NINV, neutral invertase; SPP, sucrose-phosphate phosphatase; SPS, sucrose-phosphate synthase; SUC, sucrose carrier or transporter; SUSY, sucrose synthase; UDPG, UDP-D-glucose; AINV, vacuolar acid invertase; VGT, vacuole glucose transporter; ERD6, ERD6-like transporters. STP, Sugar Transport Protein; SUC, Sucrose transporter; TMT, tonoplast membrane transporter. Abbreviations of enzymes for TCA cycle and cytoplasmic malate metabolism: PK, pyruvate kinase; PDC, pyruvate dehydrogenase complex; PDH, pyruvate dehydrogenase; CS, citrate synthase; IDH, isocitrate dehydrogenase; OGDH, 2-oxoglutarate dehydrogenase; SCS, succinyl-CoA synthetase; SDH, succinate dehydrogenase; FUM, Fumarase; cMDH, cytosolic malate dehydrogenase; NAD-ME, NAD-malic enzyme; NADP-ME, NADP-malic enzyme; PEPC, phosphoenolpyruvate carboxylase.

It is known that the inter-cellular allocation of sugars across the plasma membrane is facilitated by a serial of sucrose transporters, such as sucrose transporter (ZjSUC), although their intra-cellular distribution is conducted by vacuolar glucose transporters (ZjVGT), tonoplast membrane transporter (TMT), plastidic glucose transporter (ZjpGlcT), and ERD6-like sugar transporters (Wei et al., 2014; Zhang et al., 2018). In this study, we identified 35 proteins involved in the sugar transport and Suc-Suc cycles (Figure 6A and Supplementary Table 4). As shown in Figure 6B, these proteins were clustered into five groups based on their expression abundance and dynamics. According to the accumulation pattern of sugars, proteins in Group IV, such as ZjSTP2, 8, ZjSUS1,3, ZjTMT2, ZjSPS1,2,3, ZjSUC, and ZjERD6L-17, were upregulated during 60–100 DAF in both accessions, and their expression abundance was generally higher in ‘Junzao,’ which might promote sugar accumulation and synthesis in ‘Junzao.’ Some proteins in Group III, such as STP1,3,9 10, and ZjNINV1 were upregulated in ‘Qingjiansuanzao’ during 80–100 DAF while they were lowly expressed in ‘Junzao,’ suggesting that they might take part in the accumulation of glucose and fructose. In addition, we found that ZjAINV2,3 were highly expressed at 60 DAF in ‘Junzao’ while they maintained at low levels in ‘Qingjiansuanzao,’ which were similar to the changing pattern of enzyme AINV activity. The abundances of ZjNINV1,2 were decreased during fruit ripening in ‘Junzao’ while it was gradually increased in ‘Qingjiansuanzao’. Therefore, ZjNINVs might have regulated glucose and fructose accumulation in ‘Qingjiansuanzao’.

A total of 30 proteins were identified to be involved in malate metabolism and the TCA cycle (Figures 6A,C and Supplementary Table 4). As shown in Figure 6C, most proteins in the citrate cycle showed an increasing trend during fruit ripening, including two citrate synthesis (ZjCS), three NADP-dependent isocitrate dehydrogenase (ZjNADP-IDH), 2-oxoglutarate dehydrogenase (OGDH), succinyl-CoA synthetase (SCS1,2), succinate dehydrogenase (ZjSDH1,3), and NAD-malate dehydrogenase (ZjMDH1). The other proteins, such as ZjSDH2, fumarate hydratase (ZjFUM), and ZjSCS3, were expressed with an opposite trend. In cytosolic malate metabolism, the abundance of three phosphoenolpyruvate carboxylase (ZjPEPCs) was generally increased during fruit development and was higher in ‘Qingjiansuanzao’. For malic enzymes (ZjNAD-ME1,2 and ZjNADP-ME1,3), their expression was generally decreased during fruit ripening while ZjNADP-ME3 showed an increasing trend in both accessions. Cytosolic malate dehydrogenases (ZjcMDH3,4,5) in both accessions were upregulated during 60–100 DAF and were more abundant in ‘Qingjiansuanzao,’ consistent with the increasing trends of malic acid contents (Figure 3A).

### Differentially Expressed Proteins Involved in Ascorbic Acid Metabolism

**FIGURE 7:**
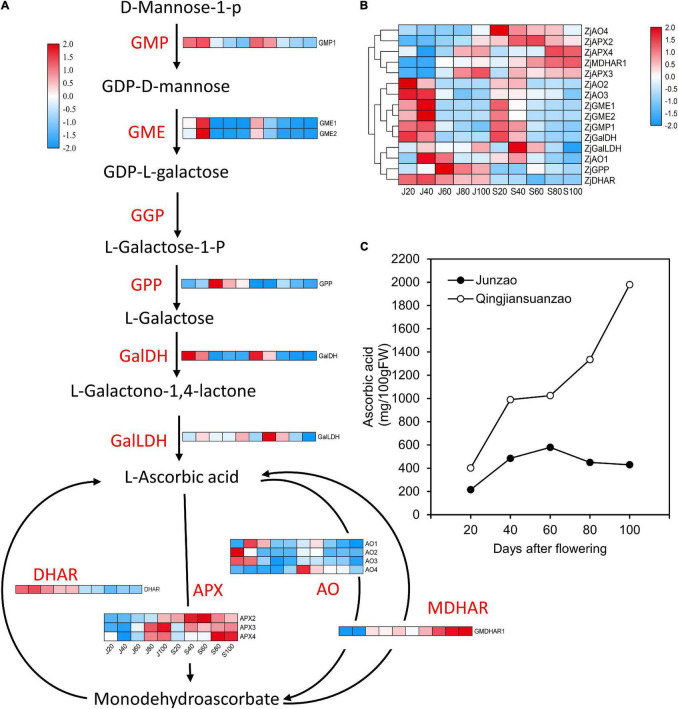
Expression profile of proteins involved in ascorbic acid (AsA) metabolism and the dynamics of AsA contents during fruit development and ripening. **(A)** Protein expression changes involved in AsA biosynthesis and recycling pathways in jujube fruits. **(B)** Cluster analysis of proteins involved in AsA metabolism. **(C)** The AsA contents in jujube fruit development. GMP, GDP-d-mannosepyrophosphorylase; GME, GDP-mannose-3′-5′-epimerase; GGP, GDP-l-galactosetransferase; GPP, l-galactose-1-phosphatephosphatase; GalDH, L-galactose dehydrogenase; GalLDH, L-galactono-1,4-lactone dehydrogenase; APX, ascorbate peroxidase; AO, ascorbate oxidase; DHAR, dehydroascorbate reductase; MDHAR, monodehydroascorbate reductase.

As shown in Figure 7C, the ascorbic acid (AsA) contents were strikingly higher in ‘Qingjiansuanzao’ than in ‘Junzao’ during fruit development. Specifically, the AsA contents were increased during fruit ripening and eventually reached 1,980.1 mg/100g in ‘Qingjiansuanzao’ while they peaked at 60 DAF and then declined to 430.5 mg/100g at 100 DAF in ‘Junzao.’ We identified 15 proteins involved in the L-galactose pathway while no proteins were identified in the myo-inositol pathway (Figure 7A and Supplementary Table 4). We also found that proteins that are related to the AsA synthesis pathway showed a downward trend during fruit development, except l-galactose-1-phosphatephosphatase (GPP) that was upregulated at 60 DAF and was higher in ‘Junzao’ than in ‘Qingjiansuanzao’. Protein GalDH peaked at 40 DAF in both accessions and had a higher abundance in ‘Qingjiansuanzao’ than in ‘Junzao.’ In the recycling metabolic pathways, the expression of ZjAOs and DHAR declined generally during fruit development and ripening in ‘Junzao’ and ‘Qingjiansuanzao’. In contrast, the expression of ZjAPXs and MDHAR1 increased generally, and in particular, the expression of ZjMDHAR1 and ZjAPX4 in ‘Qingjiansuanzao’ fruit was elevated substantially at 80 DAF, which was consistent with the AsA accumulation pattern in ‘Qingjiansuanzao’ (Figure 7B). Taken together, the L-galactose pathway is the dominant biosynthesis pathway of AsA, and the higher expression of GalDH, ZjAPXs, and MDHAR1 might contribute to the high content of AsA in ‘Qingjiansuanzao’.

## Discussion

### Jujube Fruit Expansion and the Role of Phytohormones

The domestication of fruit species is often coupled with a dramatic increase in fruit size. The cultivated jujube cultivars were derived from a single progenitor wild species-wild jujube. The long history of domestication and selection has generated many jujube cultivars of markedly enlarged fruit. Using anatomical observation, we revealed that the cell division phase in ‘Junzao’ was active through the 7th week after flowering while it ended at 4th week after flowering in ‘Qingjiansuanzao,’ suggesting that the mesocarp cell division duration in ‘Junzao’ was significantly longer and more active than in ‘Qingjiansuanzao’. However, the cell size in the mesocarp of ‘Junzao’ was only three times that in the ‘Qingjiansuanzao’ fruit. Prolonged cell production was also reported in apple fruits, where cell division continued until 70 DAF although the fruit growth after 24 DAF was primarily driven by cell expansion (Malladi and Johnson, 2011). Moreover, a study in pears concluded that the evolution of fruit size from wild *Pyrus* to cultivated *Pyrus pyrifolia* mainly resulted from shifts in the ability of cells to divide rather than to enlarge, and the final fruit size is directly related to the number of cells produced in the period immediately following pollination (Zhang et al., 2006). Interestingly, we also found that the intercellular spaces in the mesocarp accounted for much space of the fruit, and were increased during jujube fruit development. It is possible that such large intercellular spaces may enable the fruit to absorb an excessive amount of water that could break the flesh, explaining why the cultivated jujube cultivars are susceptible to rain-induced fruit crack in mature stages.

Hormones are key regulators of fruit development and ripening (McAtee et al., 2013). It is well established that auxins, GA, and cytokinin are involved in stimulating the growth of flesh tissues and determining the fruit size. In this study, we revealed that the concentration of GA_3_, IAA, and ZT in the fruit flesh was significantly higher in ‘Junzao’ than in ‘Qingjiansuanzao’ in most stages during fruit development. Studies have suggested that once the cell division stage is over, auxin and GA would become the main regulators in the cell expansion phase (Kumar et al., 2014). We found that GA_3_ was strikingly higher in ‘Junzao’ than in wild jujube. Remarkably, we also found that the expression of several important proteins, such as GA20OX and GA2OX, were much higher in ‘Junzao’ than in ‘Qingjiansuanzao,’ and G20OX, in particular, was identified as a hub protein in the Lightyellow module that was correlated with the expansion stage (J40–40 DAF) of ‘Junzao’ by WCGNA analysis. Lately, it was reported that the expression level of GA20OX in soybean seeds was correlated with seed weight, and was significantly higher in cultivated soybeans than in wild soybean. As such, GA20OX has been regarded as a key driver for seed trait formation during the domestication of soybeans (Lu et al., 2016; Gao et al., 2018). Therefore, it is possible that G20OX might be a critical protein for enlarging jujube fruit size during domestication.

### Protein Signatures Related to Fruit Expansion

In this study, the WGCNA analysis identified coexpression protein modules correlated with protein profiles at specific stages of fruit development. We found that the modules correlated with fruit expansion stages in ‘Junzao’ contained much more cell division and expansion-related proteins than those in ‘Qingjiansuanzao,’ suggesting more active cell production and expansion in ‘Junzao’ fruit. A co-expression network study in chickpea also concluded that the period of cell division during embryogenesis determined seed size based on the fact that there were cultivar-specific molecular signatures associated with seed development and seed size/weight (Garg et al., 2017). In studies focusing on the proteomic and transcriptomic profiling of apple fruit development, the proteins involved in cell division, such as DNA synthesis, RNA processes, and protein synthesis, were similarly found to be expressed at high levels during early developmental stages (Malladi and Johnson, 2011; Li et al., 2016).

In addition, we identified a series of differentially expressed proteins involved in cell production and cell wall reconstruction during fruit development. Among them, some proteins are involved in cell wall loosening, including expansions, XTHs, endoglucanases, and the proteins for pectin modifications. In apples, XTH9, expansin, and some PGs were highly expressed in ‘Greensleeves’ apple flesh during expansion at 16 DAB (Li et al., 2016). Cellulose synthase-like proteins may participate in the biosynthesis of non-cellulosic polysaccharides of the plant cell wall. We found that the ZjCSLGs abundances increased during fruit development, but the abundance of ZjCSLAs showed a decreasing trend. Similar results were observed in apples, which may be due to the different subgroups of proteins having specific biological functions (Li et al., 2016). The proteins involved in cell expansion and cell wall loosening were expressed higher and longer in ‘Junzao’ than in ‘Qingjiansuanzao,’ and therefore they likely promoted fruit expansion. Previous studies in apple reported that several B-type cyclin-dependent kinases (CDKs) were positively associated with cell production during apple fruit development (Malladi and Johnson, 2011; Li et al., 2016). In this study, we also found that proteins ZjCDKs and tubulins (ZjTUBs) showed higher expression levels in young fruits in both accessions and especially more abundant in ‘Junzao,’ thereby activating cell division and affecting the volume of jujube fruits.

### Accumulation and Balance of Sugar and Acids

This study uncovered that the pathways of sucrose and sugar metabolism enriched in the Brown and Yellow co-expression modules that were positively correlated with the protein profile at 40 DAF of ‘Junzao,’ suggesting higher activities of sugar metabolism in ‘Junzao’ than in ‘Qingjiansuanzao’ (Figure 4). Furthermore, we found that SPS had a distinct enzyme activity pattern between the two accessions, and surprisingly, SPS was also shown to be a hub protein in the WGCNA analysis. These findings appear to suggest that SPS might play a vital role in the final difference in sugar content between the cultivated and wild jujubes. Similar results were observed in citrus fruits, where sucrose is rapidly accumulated in citrus juice sac cells during the late stage of citrus fruit development, and coincidently, SPS expression was also enhanced (Katz et al., 2011). At the late stage of apple fruit development, sucrose accumulation is enhanced, consistent with the elevated expression of MdSPS5 and MdSPS6 (Li et al., 2012).

Both cultivated and wild jujubes mainly accumulate sucrose, but a much higher ratio of sucrose/glucose or fructose was observed in ‘Junzao’ than in wild jujube. By proteomics analyses, we found that the ZjAINV1 and ZjNINVs abundance change patterns were correlated with their corresponding enzyme activities and sugar contents in both accessions, suggesting that NINV may play an important role in regulating sugar components. Li et al. (2018) reported that the antisense suppression of aldose-6-phosphate reductase in apples resulted in a significantly elevated sucrose concentration in the leaves. They further found that the activities of neutral invertase and the transcript levels of MdNIV1/3 were upregulated in the transgenic fruit, indicating that NINV played an important role in maintaining the level of fructose (Li et al., 2018). In sugarcane, NINV activity was negatively correlated with sucrose content in the bottom of the internodes of sugarcane (Rose and Botha, 2000). Furthermore, the downregulation of NINV activity in sugarcane cell suspension cultures leads to an increase in sucrose accumulation (Rossouw et al., 2007). During the citrus fruits shift from growth to maturation, the expression of invertases remained unchanged, while an invertase inhibitor was upregulated toward maturation, and the sucrose was also being synthesized in citrus juice sac cells during the late stage of fruit development (Katz et al., 2011). These results supported the notion that the activities of neutral invertase may play a central role in the balance of sucrose to glucose/fructose in jujube.

### Ascorbic Acid Biosynthesis in Jujubes

The AsA contents in jujube fruit are a few dozen times that in other common fruits. There are three AsA major biosynthetic pathways identified in plants: the L-galactose, Myo-inositol, and D-galacturonic acid pathways. In the present study, we only identified proteins involved in the L-galactose pathway at the proteomics level. Our whole-genome analysis showed that the L-galactose pathway is the predominant AsA biosynthesis pathway in jujube, and the high AsA concentration in jujube fruits could be attributed to the expansion of the gene family involved in this biosynthesis pathway (Liu et al., 2014). In previous studies, we revealed the presence of L-galactose, Myo-inositol, and L-galactose pathways in ‘Junzao’ at the transcript level, and suggested that the L-galactose pathway is dominant for AsA biosynthesis and the Myo-inositol pathway might play a compensatory role in maintaining AsA accumulation (Zhang et al., 2016). In this study, we demonstrated that wild jujube ‘Qingjiansuanzao’ contained up to 2,000 mg/100g AsA, which was nearly four times that in ‘Junzao’. At the proteomics level, we showed that proteins involved in the AsA recycling pathway were upregulated at late stages, which were highly correlated with the AsA accumulation pattern in wild jujube. It has been shown that L-galactono-1,4-lactone dehydrogenase (GalLDH), ascorbate peroxidase (APX), and mono-dehydroascorbate reductase (MDHAR) are the crucial genes/enzymes in AsA biosynthesis, oxidization, and recycling, respectively; and they have higher expression levels and/or enzyme activities in wild jujube, leading to higher AsA in wild jujube (Chen et al., 2016). Taken together, we conclude that the L-galactose pathway is the biosynthesis pathway of AsA in jujube fruits, and the enhanced expression of the genes involved in this pathway would elevate the AsA levels in jujube fruits.

## Conclusion

We performed a comparative analysis of the proteomes between the cultivated and wild jujubes during fruit development by integrating biological datasets from anatomical observation, quantification of phytohormones, and assays of enzyme activities associated with sugar and organic acids. Based on the results, we may conclude the following: (1) The duration and strength of cell division and expansion in the mesocarp contributed to the final difference in fruit size. (2) The higher level of GA_3_, ZT, and IAA in ‘Junzao’ could lead to increased fruit expansion. (3) The cultivated and wild jujubes are distinctive not only at the level of the proteome, but also at the level of pathways, including sugar, organic acid, and AsA metabolisms. (4) This study helps elucidate the fruit expansion and metabolism of sugars and organic acids and AsA biosynthesis.

## Data Availability Statement

The original contributions presented in the study are included in the article/Supplementary Material, further inquiries can be directed to the corresponding authors.

## Author Contributions

RC, JH, and KX conceived and designed the experiments. XC, JH, and AH performed the field sampling, physiological analyses, and bioinformatic analyses. AH performed the anatomical observation. ZM and TG took part in the bioinformatic analyses. JH, RC, and KX wrote the manuscript. All authors have read and approved the final manuscript.

## Conflict of Interest

The authors declare that the research was conducted in the absence of any commercial or financial relationships that could be construed as a potential conflict of interest.

## Publisher’s Note

All claims expressed in this article are solely those of the authors and do not necessarily represent those of their affiliated organizations, or those of the publisher, the editors and the reviewers. Any product that may be evaluated in this article, or claim that may be made by its manufacturer, is not guaranteed or endorsed by the publisher.

## Footnotes

1
http://cytoscape.org

## Supplementary Material

Supplementary Figure 1 Kyoto Encyclopedia of Genes and Genomes pathway analysis of proteins in the modules identified by weighted gene co-expression network analysis (WGCNA).

Supplementary Table 1 Proteome datasets of ‘Junzao’ jujube and wild jujube ‘Qingjiansuanzao,’ including protein abundance with three replicates.

Supplementary Table 2 Hub proteins with high connectivity in the WGCNA modules.

Supplementary Table 3 Identified proteins involved in phytohormone, cell division, expansion, and cell wall modification.

Supplementary Table 4 Identified proteins involved in sugar transportation, metabolism, acid metabolism, and ascorbic acid metabolism.
