# Supplementary figures and images for: Integrative Morphological, Physiological, Proteomics Analyses of Jujube Fruit Development Provide Insights Into Fruit Quality Domestication From Wild Jujube to Cultivated Jujube
Source: Front Plant Sci. 2021 Nov 24;12:773825. doi: 10.3389/fpls.2021.773825 (PMC8653901; doi:10.3389/fpls.2021.773825)

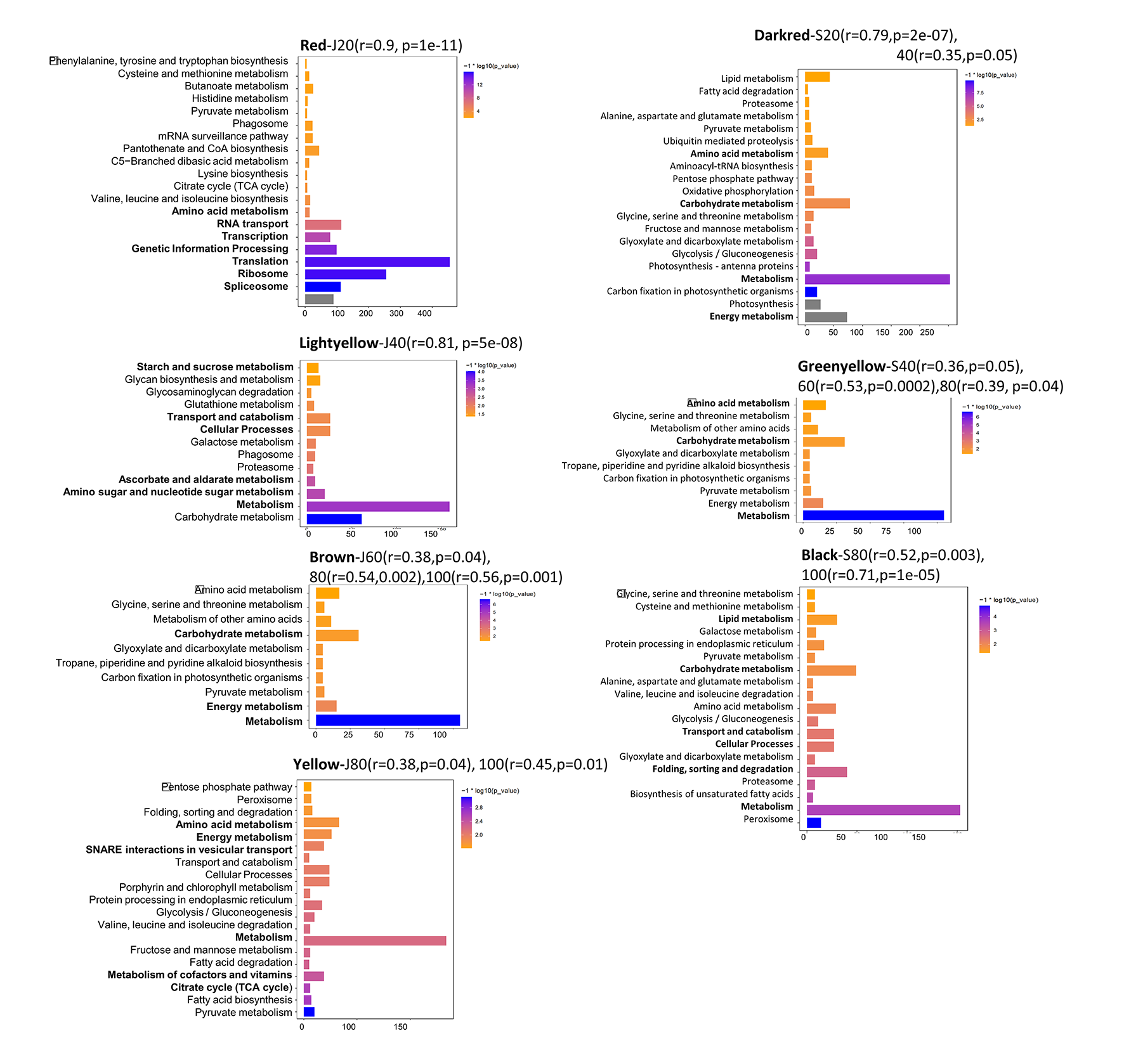

Supplement: Supplementary Figure 1 — Kyoto Encyclopedia of Genes and Genomes pathway analysis of proteins in the modules identified by weighted gene co-expression network analysis (WGCNA). [file Image_1.TIF]
